# Supplementary material for: Glucocorticoid activation of anti-inflammatory macrophages protects against insulin resistance
Source: Nat Commun. 2023 Apr 20;14:2271. doi: 10.1038/s41467-023-37831-z (PMC10119112; doi:10.1038/s41467-023-37831-z)
Supplement: Supplementary file 1 — Supplementary Information [file 41467_2023_37831_MOESM1_ESM.pdf]

Glucocorticoid Activation of Anti-Inflammatory Macrophages Protects Against Insulin Resistance

Supplementary Information

Giorgio Caratti[1, 11§], Ulrich Stifel[1§], Bozhena Caratti[1], Ali J.M. Jamil[2,3], Kyoung-Jin Chung[4], Michael Kiehntopf[5], Markus H. Gräler[6,7,8] Matthias Blüher[9], Alexander Rauch[2,10,#] and Jan P. Tuckermann[1,#,*]

[1] Institute of Comparative Molecular Endocrinology, University of Ulm, Ulm, Germany

[2] Molecular Endocrinology & Stem Cell Research Unit, Department of Endocrinology and Metabolism, Odense University Hospital, Denmark

[3]Department of Clinical Research, University of Southern Denmark, Odense, Denmark

[4]Institute for Clinical Chemistry and Laboratory Medicine, University Hospital and Faculty of Medicine, Technical University Dresden, Dresden, Germany

[5]SG Sepsis Research Clinic for Anesthesiology and Intensive Care, Jena University Hospital, Germany

[6] Department of Anesthesiology and Intensive Care Medicine, Jena University Hospital, Jena, Germany

[7]Center for Molecular Biomedicine (CMB), Jena University Hospital, Jena, Germany

[8]Center for Sepsis Control and Care (CSCC), Jena University Hospital, Germany

[9] Department of Endocrinology and Nephrology, University of Leipzig, Germany

[10] Steno Diabetes Center Odense, Odense, Denmark

[11]Current address: NIHR Oxford Biomedical Research Centre, John Radcliffe Hospital, Oxford, UK OX3 9DU and Oxford Centre for Diabetes, Endocrinology and Metabolism, University of Oxford, Oxford, OX37LE, UK

[*] Further information and requests should be directed to and will be fulfilled by the Lead Contact, Jan Tuckermann (jan.tuckermann@uni-ulm.de).

[§] These authors contributed equally

# These authors jointly supervised this work and are corresponding authors.

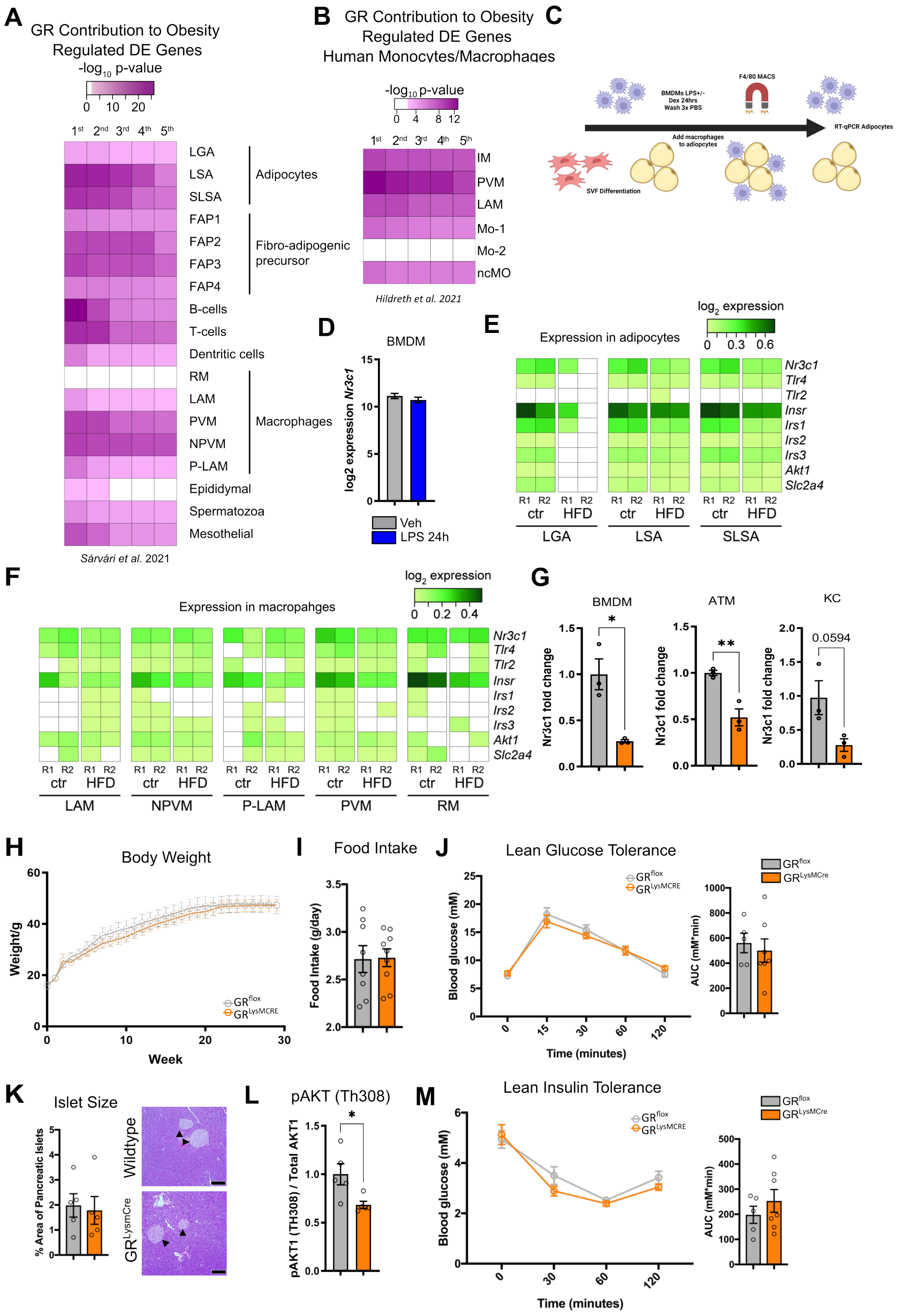

Supplementary Figure 1. Related to Figure 1

(**A**) Data from *Sárvári et al.*[1] were analysed, and genes induced by obesity were determined in each cell type and subjected to LISA analysis to estimate the prediction score of GR. fibro-adipogenic progenitors (FAPs), perivascular-like macrophages (PVM), non-perivascular-like

macrophages (NPVM), lipid-associated macrophages (LAM), proliferative LAM (P-LAM), collagen-expressing macrophages (CEM), regulatory macrophages (RM), lipogenic adipocytes (LGA), lipid-scavenging adipocytes (LSA), stressed lipid-scavenging adipocytes (SLSA). (**B**) Human monocyte and macrophage obesity regulated genes from *Hildreth et al.* [2] were analysed by LISA for GR enrichment. (**C**) Schematic representation of adipocyte-macrophage co-cultures. Created with Biorender.com (**D**) Gene expression of *Nr3c1* in BMDMs after LPS treatment. (N=2) (**E**) *Nr3c1* and *Tlr4, Tlr2, Insr, Irs1, Irs2, Irs3, Akt* and *Slc2a4* gene expression in adipocytes during chow and HFD. (**F**) *Nr3c1* and *Tlr4, Tlr2, Insr, Irs1, Irs2, Irs3, Akt* and *Slc2a4* gene expression in adipose tissue macrophages during chow and HFD (**G**) *Nr3c1* (GR) deletion was investigated via qPCR from indicated macrophage populations (BMDM = bone marrow derived macrophages, ATM = Adipose tissue resident macrophages, KC = Kupffer cells). (N=3) (**H**) GR^{flox} and $GR^{LysMCre}$ mice were subjected to 60% HFD for 29 weeks. Weight was monitored. (**I**) Food intake of mice on 60% HFD ((GR^{flox} N=8, $GR^{LysMCre}$ N=9)). (**J**) Lean GR^{flox} and $GR^{LysMCre}$ mice were fasted overnight, given 2 mg/kg glucose, and blood glucose levels were traced for 120 mins (GR^{flox} N=5, $GR^{LysMCre}$ N=7). (**K**) Pancreas was harvested from obese GR^{flox} and $GR^{LysMCre}$ mice, fixed and stained using H&E. Pancreatic islet size was quantified relative to total area of the image (N=5). (**L**) Baseline AKT phosphorylation was measured by ELISA from muscle of lean and obese GR^{flox} and $GR^{LysMCre}$ mice. (N=5) (**M**) Lean GR^{flox} and $GR^{LysMCre}$ mice were fasted overnight, and given 0.5 μ i.U./g insulin, blood glucose levels were traced for 120 mins (GR^{flox} N=5, $GR^{LysMCre}$ N=7). Original images at 10x magnification. Black arrows indicate islets. Data show mean ±SEM. Statistical analysis by student's t-test or 2-way ANOVA with a Bonferroni post-hoc test or two-tailed student's T-test *$p < 0.05$. Wilcoxon rank-sum test for A and B. Scale bar: 200μm in **K**.

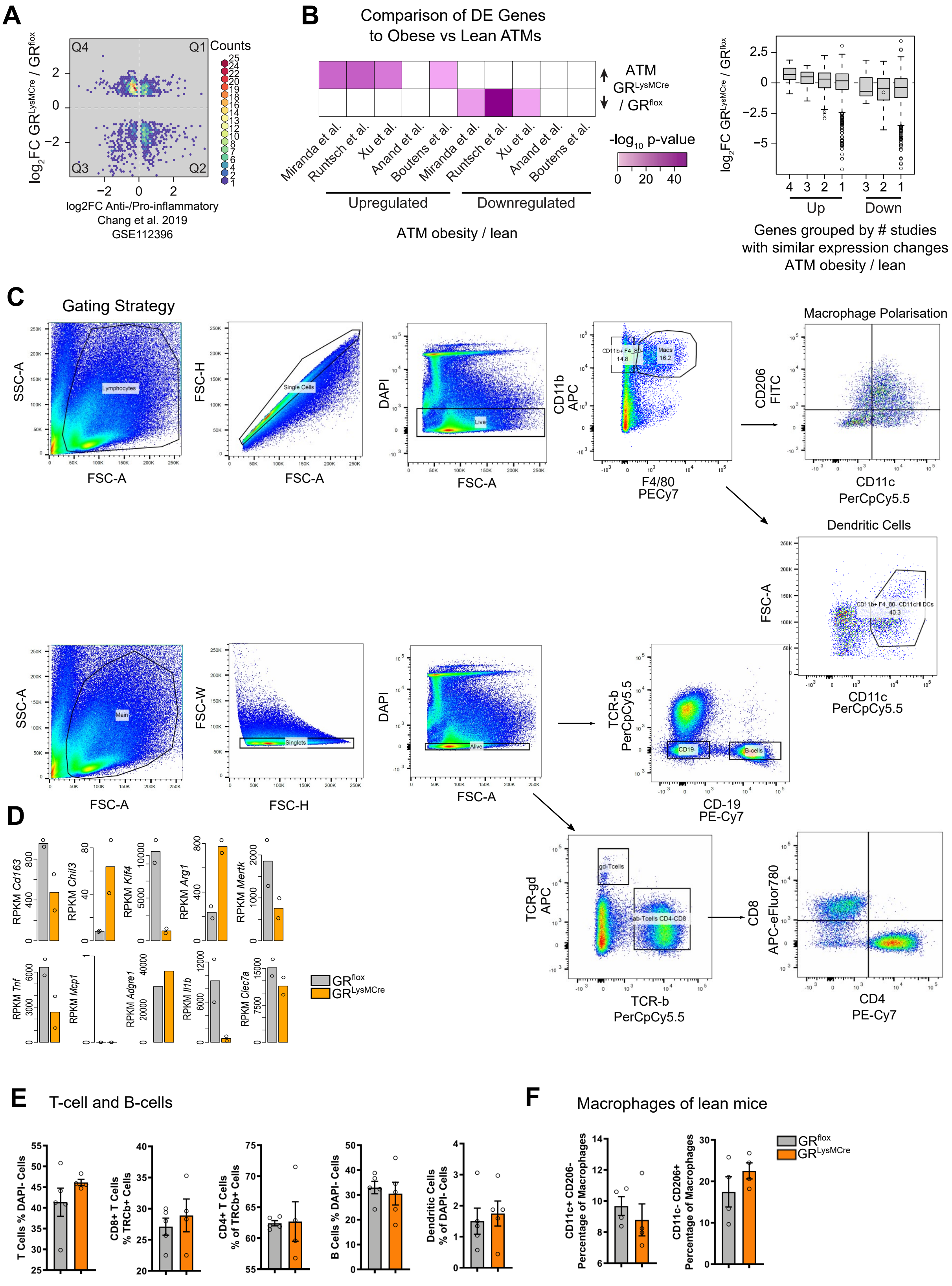

Supplementary Figure 2. Related to Figure 2.

(**A**) Scatterplot of genes differentially expressed between GR^{LysMCre} and GR^{flox} ATMs of obese mice from analysis shown in Fig. 2A compared to differentially expressed genes between anti- and pro-inflammatory ATMs from eWAT of obese mice (GSE112396)[3] . (**B**) Differentially

expressed genes between $GR^{LysMCre}$ and GR^{flox} ATMs were compared to various published datasets of ATMs between lean and obese mice. Boxplots show median, IQR, 1.5xIQR and outlier values. (N=2) (**C**) FACS gating strategy of SVF from obese GR^{flox} and $GR^{LysMCre}$ mice related to Fig.3. (**D**) Selected macrophage genes from RNASeq of RNA preparations of ATMs of obese $GR^{LysMCre}$ and GR^{flox} mice (**E**) SVF T cell relative number, polarisation, B cell relative number and dendritic cell relative number quantified by FACS (GR^{flox} N=5, $GR^{LysMCre}$ N=4). (**F**) SVF from lean GR^{flox} and $GR^{LysMCre}$ mice was analysed for macrophage polarisation by FACS (N=4). Data show mean +/- SEM. Statistical analysis by two-tailed student's t-test. $p < 0.001$***.

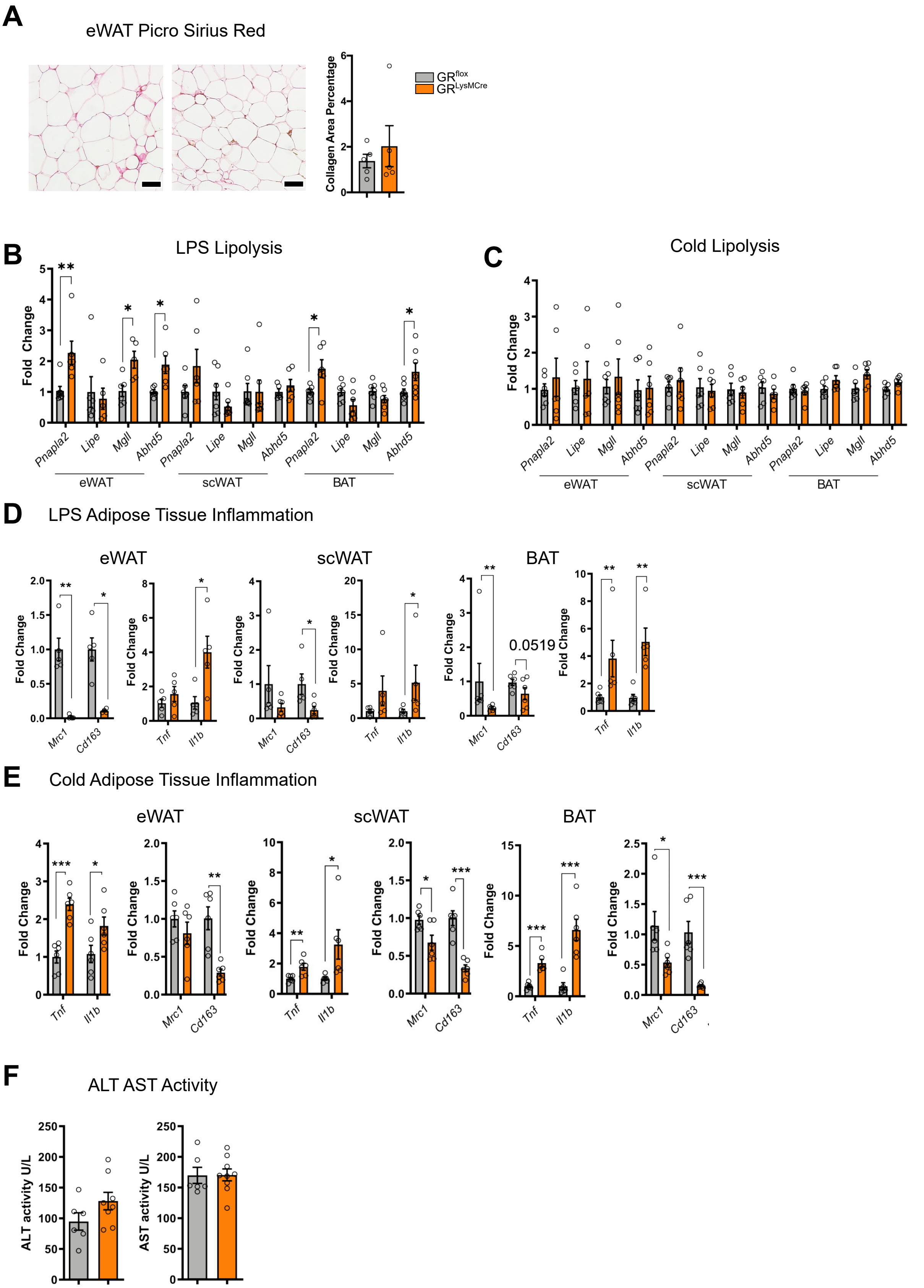

Supplementary Figure 3. Relate Figure 3

A) Picro sirius red staining of eWAT of obese GR^{flox} and GR^{LysMCre} mice (N=5). (**B**) Lean GR^{flox} and GR^{LysMCre} mice were subjected to endotoxin shock for 24 hours. RT-qPCR of lipolytic

markers (eWat GR^{flox}: *Pnpla2* N=7, *Lipe* N=6, *Mgll* N=6, *Abhd5* N=6; $GR^{LysMCre}$: *Pnpla2* N=6, *Lipe* N=5, *Mgll* N=5, *Abhd5* N=6; scWat GR^{flox}: *Pnpla2* N=6, *Lipe* N=7, *Mgll* N=7, *Abhd5* N=5; $GR^{LysMCre}$: *Pnpla2* N=6, *Lipe* N=7, *Mgll* N=7, *Abhd5* N=7; BAT GR^{flox}: *Pnpla2* N=7, *Lipe* N=7, *Mgll* N=7, *Abhd5* N=7; $GR^{LysMCre}$: *Pnpla2* N=7, *Lipe* N=7, *Mgll* N=7, *Abhd5* N=7) and (**D**) inflammation markers (eWat GR^{flox}: *Mrc1* N=5, *Cd163* N=5, *Tnf* N=5, *Il1b* N=5; $GR^{LysMCre}$: *Mrc1* N=5, *Cd163* N=5, *Tnf* N=5, *Il1b* N=5; scWat GR^{flox}: *Mrc1* N=5, *Cd163* N=5, *Tnf* N=5, *Il1b* N=5; $GR^{LysMCre}$: *Mrc1* N=6, *Cd163* N=6, *Tnf* N=5, *Il1b* N=5; BAT GR^{flox}: *Mrc1* N=6, *Cd163* N=6, *Tnf* N=6, *Il1b* N=6; $GR^{LysMCre}$: *Mrc1* N=6, *Cd163* N=6, *Tnf* N=5, *Il1b* N=6). (**C**) Obese GR^{flox} and $GR^{LysMCre}$ mice were subjected to 4°C cold stress for 12 hours. RT-qPCR of lipolytic genes (N=6) and (**E**) inflammatory markers (N=6). (**F**) Serum ALT and AST measurement of obese GR^{flox} and $GR^{LysMCre}$ mice (GR^{flox} N=6, $GR^{LysMCre}$ N=8). Data show mean +/- SEM. Statistical analysis by two-tailed Student's t-test. $p < 0.05$*, < 0.01**, <0.001***. Scale bar: 200μm in **A**.

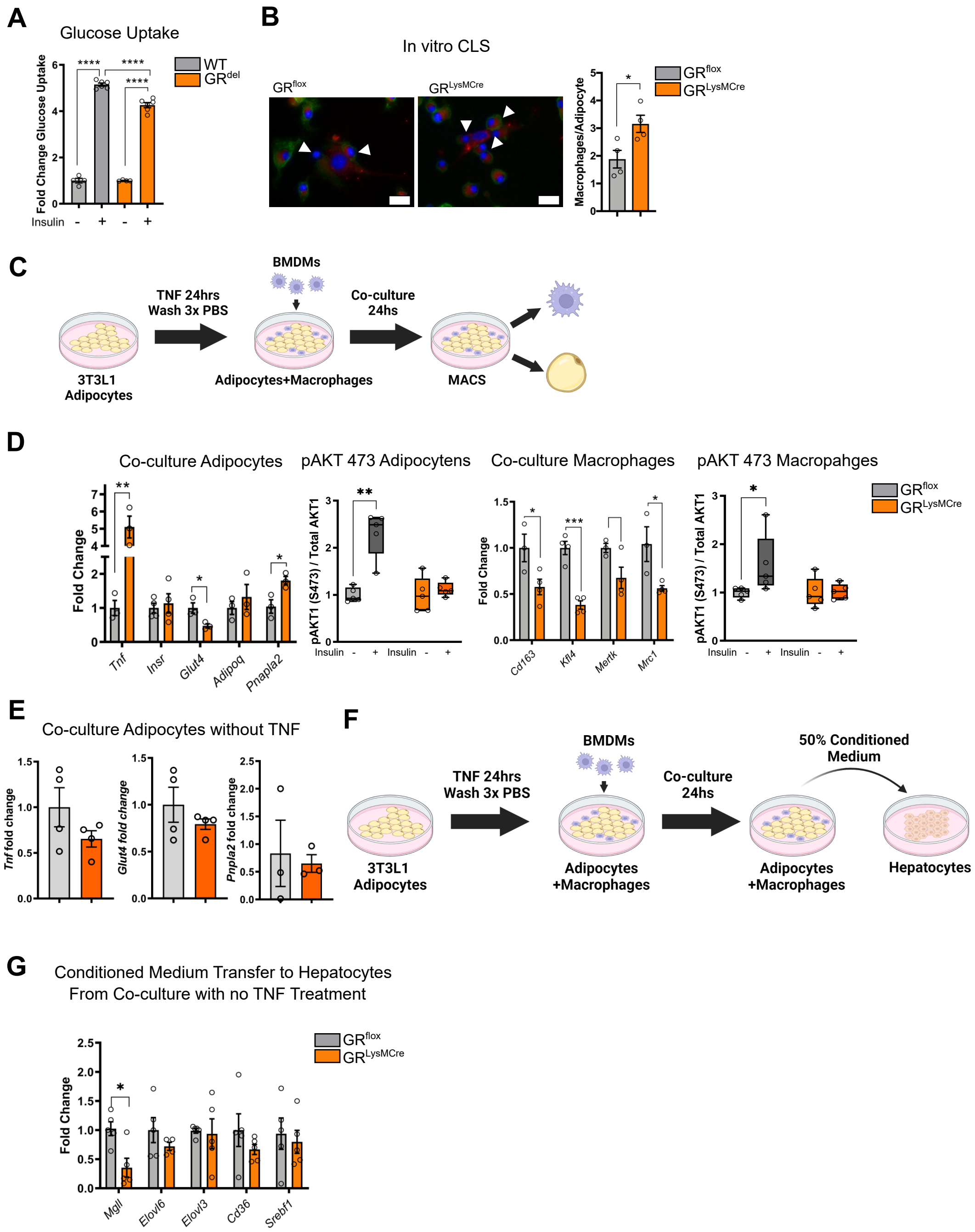

Supplementary Figure 4. Related to Figure 3.

Differentiated 3T3L1 adipocytes were co-cultured with either WT or GR^{del} fetal liver derived macrophages (FLDMs) and insulin stimulated glucose uptake assessed (WT N=4, GR^{del} N=5) (**B**) Number of GR^{flox} and GR^{LysMCre} BMDMs contacting TNF pre-treated 3T3L1 adipocytes determined by IF of CD11b (green) and LipidTox (red) (N=4). (**C**) Cartoon depicting co-cultures of TNF pre-treated 3T3L1 adipocytes and either GR^{flox} or GR^{LysMCre} BMDMs. Created with Biorender.com (**D**) 3T3L1s cocultured with either GR^{flox} or GR^{LysMCre} macrophages as in S4C were separated by MACS and gene expression or AKT phosphorylation quantified by RT-qPCR (Adipocytes: GR^{flox}: *Tnf* N=3, *Insr* N=4, *Glut4* N=3, *Adipoq* N=3, *Pnpla2* N=3; GR^{LysMCre} *Tnf* N=3, *Insr* N=4, *Glut4* N=3, *Adipoq* N=3, *Pnpla2* N=3; pAKT Adipocytes N=5; Macropahges: GR^{flox}: *Cd163* N=3, *Klf4* N=4, *Mertk* N=3, *Mrc1* N=3; GR^{LysMCre} *Cd163* N=4, *Klf4* N=4, *Mertk* N=4, *Mrc1* N=4; pAKT Macropahges N=5). (**E**) 3T3L1 adipocytes were co-cultured with BMDMs without TNF pretreatment (GR^{flox}: *Tnf* N=4, *Glut4* N=4, *Pnpla2* N=3; GR^{LysMCre}

Tnf N=4, *Glut4* N=4, *Pnpla2* N=3). (**F**) Cartoon depiction of co-culture conditioned medium transfer to hepatocytes. Created with Biorender.com (**G**) Primary hepatocytes were treated with co-culture conditioned medium without TNF pre-treatment of adipocytes (N=5). Data show mean ±SEM. Boxplots show median, IQR and min/max values. Statistical analysis two-tailed students t-test (B, D, G), two-way ANOVA with a Sidak post-hoc test (A). $p < 0.05$*, $p < 0.01$**, $p < 0.001$*** images at 40x original magnification. Scale bar: 25µm in **B**.

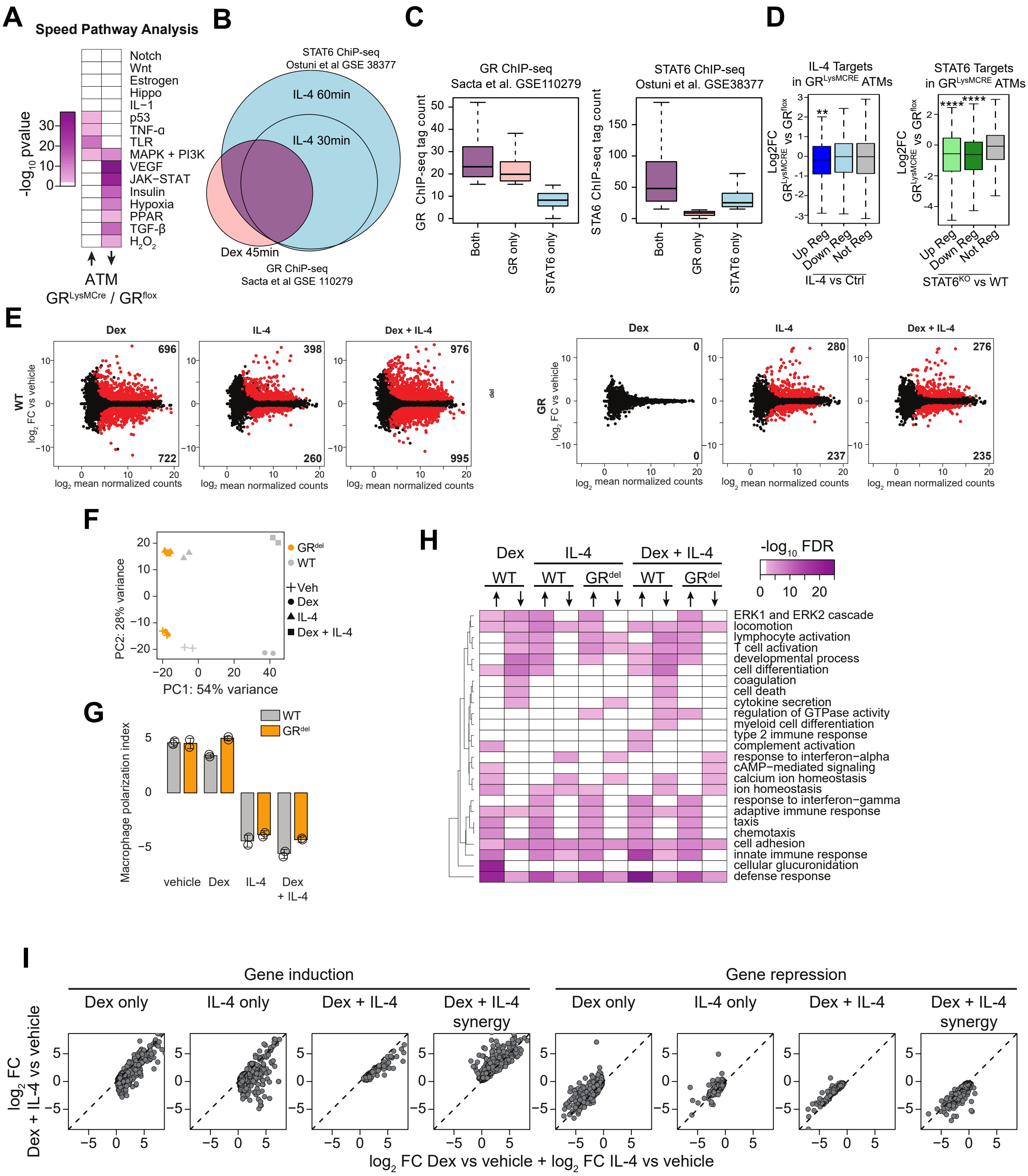

Supplementary Figure 5. Related to Figure 5.

(**A**) Speed pathway analysis of differentially expressed genes from GR^{flox} and GR^{LysMCRE} ATMs in 2A. Significance calculated using Hypergeometric test one-sided (**B**) Euler plot comparing binding loci of public ChIP-seq data for GR and STAT6. (**C**) Tag density for GR binding was determine for GR only bound, STAT6 only, GR+STAT6 bound or neither sites from the indicated studies. Tag density of STAT6 was compared for STAT6 only bound, GR only, STAT6+GR bound or neither sites from the indicated studies. (n= GR only: 2602; STAT6 only: 23665; Both: 8813) (**D**) The log$_2$FC between GR^{LysMCre} and GR^{flox} ATMs for IL-4 or STAT6^{KO} up- or down-regulated was compared to non-IL-4 or STAT6^{KO} regulated genes. Box plots show median, interquartile range and min/max values excluding outliers. P. value calculated using Wilcoxon rank sum test. (**E**) MA plots derived from RNA-seq of WT and GR^{del} FLDMs treated

with dex, IL-4 or IL-4+dex compared to vehicle. (**F**) PCA of RNA-seq data derived from S5D. (**G**) Macrophage polarization index based on RNA-seq counts from WT and GR^{del} FLDMs in S5D. (N=2) (**H**) Gene ontology analysis of genes up or down regulated by dex (only WT, no genes regulated in GR^{del}), IL-4 and dex+IL4 compared to vehicle control (GOseq benjamini-hochberg corrected padj < 0.01). (**I**) Scatter plots of quantification of dex+IL-4 synergism comparing log_2FC of dex+IL-4 to log2FC dex + log2FC IL-4. Exact p.value: D: IL4 targets: 0.0050, Stat6 targets: Up-regulated: 6.849e-10, Down-regulated: <2.2e-16

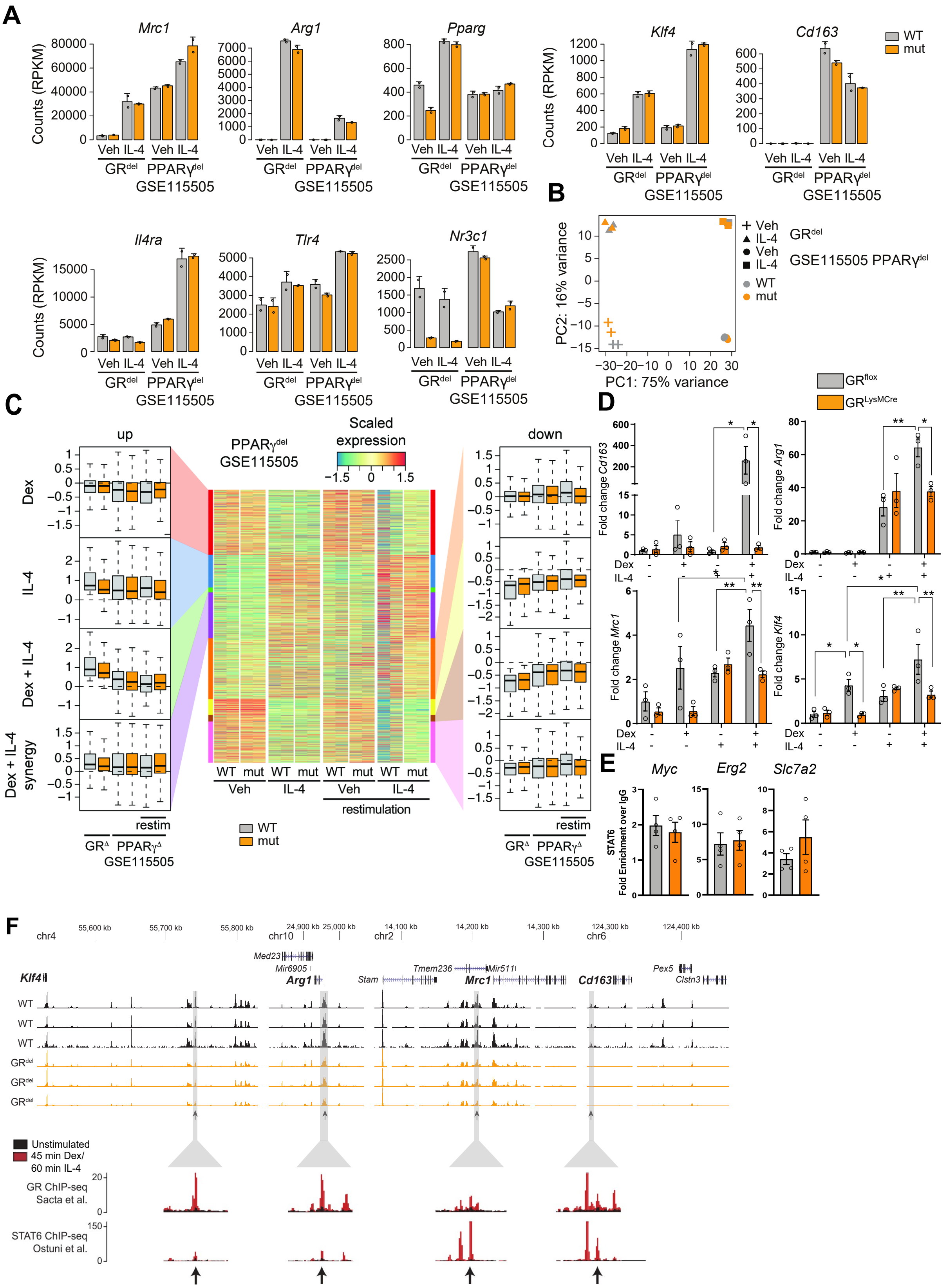

Supplementary Figure 6 Related to Figure 5.

(**A**) Gene expression from GR^{del} and PPARγ^{del} BMDMs with and without IL-4 treatment. (**B**) Principal component analysis for the GR^{del} and PPARγ^{del} BMDMs before and after stimulation

with IL-4. (**C**) Heat map showing gene expression of first and second stimulation with IL-4 in PPARγ^{del} BMDMs for the clusters identified in Figure 4B along with boxplots quantifying the induction and repression levels for GR and PPARγ deficiency. (**D**) qPCR validation of dex+IL-4 co-regulated genes (N=3). (**E**) ChIP PCR for STAT6 in GR^{flox} and GR^{LysMCre} BMDMs upon dex + IL-4 stimulation nearby genes of the IL-4 only activated group (N=4). (**F**) Design for the ChiP-qPCR Primers for their respective genes. Data show mean ±SEM. Boxplots show median, IQR and min/max values. Statistical analysis by two-way ANOVA (D), Mann-Whitney test, two-sided (E). $p < 0.05$*, $p < 0.01$**, $p < 0.001$***.

Supplementary References

1. Sárvári, A. K. *et al.* Plasticity of Epididymal Adipose Tissue in Response to Diet-Induced Obesity at Single-Nucleus Resolution. *Cell Metabolism* **33**, 437-453.e5 (2021).

2. Hildreth, A. D. *et al.* Single-cell sequencing of human white adipose tissue identifies new cell states in health and obesity. *Nat Immunol*. **22**:639-653 (2021).

3. Chang, J. C. et al. Adaptive adipose tissue stromal plasticity in response to cold stress and antibody-based metabolic therapy. Sci Rep **9**, 8833 (2019).

Supplementary Table 1

RT-qPCR Primer

Name	Forward	Reverse	
Abhd5	TGGTGTCCCACATCTACATCA	CAGCGTCCATATTCTGTTTCCA	
Adgre1	CCTGGACGAATCCTGTGAAG	GGTGGGACCACAGAGAGTTG	
Adipoq	GGAGAGAAAGGAGATGCAGGT	CTTTCCTGCCAGGGGTTC	
Arg1	CTCCAAGCCAAAGTCCTTAGAG	AGGAGCTGTCATTAGGGACATC	
Cd163	GGCTAGACGAAGTCATCTGCA	CTTCGTTGGTCAGCCTCAGAGA	
Cd36	TGGCAAAGAACAGCAGCAAA	CACAGTGTGGTCCTCGGG	
Chil3	GCTCTCCAGAAGCAATCCTGA	GGAAGATCCCAGCTGTACGTT	
Clec7a	CTTCACCTTGGAGGCCCATT	CGCCAAAATGCTAGGGCAC	
Elovl3	TTCTCACGCGGGTTAAAAATGG	GAGCAACAGATAGACGACCAC	
Elovl6	TCAGCAAAGCACCCGAAC	AGCGACCATGTCTTTGTAGGAG	
Glut4	CTGTCGCTGGTTTCTCCAAC	CAGGAGGACGGCAATAGAA	
Il1b	GGCTGTGGAGAAGCTGTGGCA	GGGTCCGACAGCACGAGGCT	
Insr	TCTTTCTTCAGGAAGCTACATCTG	TGTCCAAGGCATAAAAAGAATAGTT	
Klf4	GCAGTCACAAGTCCCCTCTC	TGGTAAGGTTTCTCGCCTGT	
Lipe	CCAGCCTGAGGGCTTACTG	CTCCATTGACTGTGACATCTCG	
Mcp1	AACGCCCCACTCACCTGCTG	TGGGGTCAGCACAGACCTCTC	
Mertk	GCTGGCATTTCATGGTGGAA	CATTGTCTGAGCGCTGCAC	
Mgll	ACCATGCTGTGATGCTCTCTG	CAAACGCCTCGGGGATAACC	
Mrc1	CCACAGCATTGAGGAGTTTG	ACAGCTCATCATTTGGCTCA	
Nr3c1	GGCCGCTCAGTGTTTTCTAA	GCAGAGTTTGGGAGGTGGT	
Pnpla2	CAACGCCACTCACATCTACGG	GGACACCTCAATAATGTTGGCAC	
Rpl	CCTGCTGCTCTCAAGGTT	TGGCTGTCACTGCCTGGTACTT	
Srebf1	TGACCCGGCTATTCCGTGA	CTGGGCTGAGCAATACAGTTC	
Tnf	AGGGGCCACCACGCTCTTCT	TGAGTGTGAGGGTCTGGGCCAT	

ChIP Primer

Arg1	CAAAGGGTTCTGCAGGGGAA	TCAAGAGCCCTAAGTGCCTG	
Cd163	CTCAGTTGGGGTGAGACTTCA	TGCAAGCTCTAGCCAGACAAC	
Erg2	CAAATTGGCCATGTGACGGC	GCAGGTCTTCAATACCCGTGA	
Klf4	GGTGACAAAGCAGGTGAAGC	TGTACAAAGCCGGTCTAGCG	
Mrc1	TCCAGGTCCATCCATTTGGC	TGGAAAGAACCCAGATGCCC	
Myc	TTGGCAATTCACTCCTCCCC	GCGCTAGACGCGAGAATATG	
Slc7a2	AGCTTGGAGTTAGACACCGC	CCTGGAGCGTTTGTCACGG	
