# Supplementary material for: Glucocorticoid activation of anti-inflammatory macrophages protects against insulin resistance
Source: Nat Commun. 2023 Apr 20;14:2271. doi: 10.1038/s41467-023-37831-z (PMC10119112; doi:10.1038/s41467-023-37831-z)
Supplement: Supplementary file 3 — Reporting Summary [file 41467_2023_37831_MOESM3_ESM.pdf]

nature portfolio

Corresponding author(s): Alexander Rauch
Jan Tuckermann

Last updated by author(s): Mar 31, 2023

Reporting Summary

Nature Portfolio wishes to improve the reproducibility of the work that we publish. This form provides structure for consistency and transparency in reporting. For further information on Nature Portfolio policies, see our Editorial Policies and the Editorial Policy Checklist.

Statistics

For all statistical analyses, confirm that the following items are present in the figure legend, table legend, main text, or Methods section.

n/a	Confirmed	
☐	☒	The exact sample size (*n*) for each experimental group/condition, given as a discrete number and unit of measurement
☐	☒	A statement on whether measurements were taken from distinct samples or whether the same sample was measured repeatedly
☐	☒	The statistical test(s) used AND whether they are one- or two-sided<br>*Only common tests should be described solely by name; describe more complex techniques in the Methods section.*
☒	☐	A description of all covariates tested
☐	☒	A description of any assumptions or corrections, such as tests of normality and adjustment for multiple comparisons
☐	☒	A full description of the statistical parameters including central tendency (e.g. means) or other basic estimates (e.g. regression coefficient) AND variation (e.g. standard deviation) or associated estimates of uncertainty (e.g. confidence intervals)
☐	☒	For null hypothesis testing, the test statistic (e.g. *F*, *t*, *r*) with confidence intervals, effect sizes, degrees of freedom and *P* value noted<br>*Give P values as exact values whenever suitable.*
☒	☐	For Bayesian analysis, information on the choice of priors and Markov chain Monte Carlo settings
☒	☐	For hierarchical and complex designs, identification of the appropriate level for tests and full reporting of outcomes
☒	☐	Estimates of effect sizes (e.g. Cohen's *d*, Pearson's *r*), indicating how they were calculated

Our web collection on statistics for biologists contains articles on many of the points above.

Software and code

Policy information about availability of computer code

Data collection: Published micro array expression data were collected using GEO2R and sequencing data were downloaded fastq-dump from SRA.

Data analysis:
HOMER http://homer.salk.edu/homer/
goseq https://bioconductor.org/packages/release/bioc/html/goseq.html
Bedtools http://bedtools.readthedocs.io/en/latest/
STAR aligner https://github.com/alexdobin/STAR
DEseq2 https://bioconductor.org/packages/release/bioc/html/DESeq2.html
LISA http://lisa.cistrome.org/
In addition we utilized GraphPad Prism 9 ,FlowJo_V10 and Image J 1.53t Fiji

For manuscripts utilizing custom algorithms or software that are central to the research but not yet described in published literature, software must be made available to editors and reviewers. We strongly encourage code deposition in a community repository (e.g. GitHub). See the Nature Portfolio guidelines for submitting code & software for further information.

Data

Policy information about availability of data

All manuscripts must include a data availability statement. This statement should provide the following information, where applicable:

- Accession codes, unique identifiers, or web links for publicly available datasets
- A description of any restrictions on data availability
- For clinical datasets or third party data, please ensure that the statement adheres to our policy

Data availability statement:
Raw sequencing and DESeq2 processed data of the ChIP and RNA-seq experiments generated in this work are deposited under the accession number: GSE200371 (https://www.ncbi.nlm.nih.gov/geo/query/acc.cgi?acc=GSE200371).
Other datasets used:
GSE160729 (https://www.ncbi.nlm.nih.gov/geo/query/acc.cgi?acc=GSE160729)
GSE167382 (https://www.ncbi.xyz/geo/query/acc.cgi?acc=GSE167382)
GSE112396 (https://www.ncbi.nlm.nih.gov/geo/query/acc.cgi?acc=GSE112396)
GSE119703 (https://www.ncbi.nlm.nih.gov/geo/query/acc.cgi?acc=GSE119703)
GSE53403 (https://www.ncbi.nlm.nih.gov/geo/query/acc.cgi?acc=GSE53403)
GSE84000 (https://www.ncbi.nlm.nih.gov/geo/query/acc.cgi?acc=GSE84000)
GSE63171 (https://www.ncbi.nlm.nih.gov/geo/query/acc.cgi?acc=GSE63171)
GSE114735 (https://www.ncbi.nlm.nih.gov/geo/query/acc.cgi?acc=GSE114735)
GSE112396 (https://www.ncbi.nlm.nih.gov/geo/query/acc.cgi?acc=GSE112396)
GSE115505 (https://www.ncbi.nlm.nih.gov/geo/query/acc.cgi?acc=GSE115505)
GSE25088 (https://www.ncbi.nlm.nih.gov/geo/query/acc.cgi?acc=GSE25088)
GSE151015 (https://www.ncbi.nlm.nih.gov/geo/query/acc.cgi?acc=GSE151015)
GSE110279 (https://www.ncbi.nlm.nih.gov/geo/query/acc.cgi?acc=GSE110279)
GSE38377 (https://www.ncbi.nlm.nih.gov/geo/query/acc.cgi?acc=GSE38377)

Human research participants

Policy information about studies involving human research participants and Sex and Gender in Research.

Reporting on sex and gender	N/A
Population characteristics	N/A
Recruitment	N/A
Ethics oversight	N/A

Note that full information on the approval of the study protocol must also be provided in the manuscript.

Field-specific reporting

Please select the one below that is the best fit for your research. If you are not sure, read the appropriate sections before making your selection.

☒ Life sciences ☐ Behavioural & social sciences ☐ Ecological, evolutionary & environmental sciences

For a reference copy of the document with all sections, see nature.com/documents/nr-reporting-summary-flat.pdf

Life sciences study design

All studies must disclose on these points even when the disclosure is negative.

Sample size	Animal experiments were designed based with calculations to achieve 80% power with 5% type 1 error. Cell culture experiments had no formal sample size calculation. Sample size determination here was based on previously published work (Stifel, Mol.Met 2022, Mueller, Diabetes 2017)
Data exclusions	Outliers detected using a ROUT test in graphpad prism were excluded from RT-qPCR analyses.
Replication	The HFD feeding experiment represent 2-3 independent experiments. All attempts at replication were successful.<br>In vitro work was completed with a minimum of 2 biological replicates from independent experiments. All attempts at replication were successful.

Randomization	Animals could not be randomized due to genotype requirements. However each cage housed both genotypes and all animals, dependent on genotype were random allocated to a lean or HFD feeding regimen
Blinding	No formal blinding was done during sample collection as the genotypes of the animals were marked on the cages. Analysis of histology, qPCR and other in vitro experiments was performed blinded.

Reporting for specific materials, systems and methods

We require information from authors about some types of materials, experimental systems and methods used in many studies. Here, indicate whether each material, system or method listed is relevant to your study. If you are not sure if a list item applies to your research, read the appropriate section before selecting a response.

Materials & experimental systems

n/a	Involved in the study	
☐	☒	Antibodies
☐	☒	Eukaryotic cell lines
☒	☐	Palaeontology and archaeology
☐	☒	Animals and other organisms
☒	☐	Clinical data
☒	☐	Dual use research of concern

Methods

n/a	Involved in the study	
☐	☒	ChIP-seq
☐	☒	Flow cytometry
☒	☐	MRI-based neuroimaging

Antibodies

Antibodies used

Mouse monoclonal H3K27ac antibody Active Motif #39685; RRID: AB_2793305 5µg (IP)
anti-CD45 coupled beads 10µl/sample
anti-CD16/32 (block) 1/300
anti-F4/80, coupled beads 10µl/sample
anti-CD11b FITC 1/300
anti-GR antibody 3µg (IP)
anti-STAT6 1/50 (IP)
TCRb PerCpCy5.5 1/400
TCRgd APC 1/400
CD4 PeCy7 1/400
CD8 APC-eFluor 780 1/400
CD11c PerCpCy5.5 1/400
CD19 PeCy7 1/400
CD11b PerCpCy5.5 1/400
CD11b APC 1/400
CD11b AF488 1/400
F4/80 PeCy7 1/400
CD206 FITC 1/400
anti-F4/80 (SCBT) 1/100
anti-F4/80 (CellSignaling) 1/100
anti-CD206 1/300
anti-CD11c 1/200

Validation

The antiboidies used in this study are validated by their respective manufacturers and are commercially available:
H3K27ac: https://ngsqc.org/cert_reports/Hela_H3K27ac_Homo%20sapiens_Active%20Motif_39685_16611003_monoclonal.pdf
anti-CD45 MicroBeads https://www.miltenyibiotec.com/DE-en/products/cd45-microbeads-mouse.html
anti-CD16/32 https://www.thermofisher.com/antibody/product/CD16-CD32-Antibody-clone-93-Monoclonal/14-0161-82
anti-F4/80, Miltenyi https://www.miltenyibiotec.com/DE-en/products/anti-f4-80-microbeads-ultrapure-mouse.html
anti-CD11b FITC https://www.thermofisher.com/antibody/product/CD11b-Antibody-clone-ICRF44-Monoclonal/11-0118-42
anti-GR antibody (IP) https://www.ptglab.com/products/NR3C1-Antibody-24050-1-AP.htm
anti-STAT6 (IP) https://www.cellsignal.com/products/primary-antibodies/stat6-d3h4-rabbit-mab/5397
TCRb PerCpCy5.5 https://www.thermofisher.com/antibody/product/TCR-beta-Antibody-clone-H57-597-Monoclonal/45-5961-82
TCRgd APC https://www.thermofisher.com/antibody/product/TCR-gamma-delta-Antibody-clone-eBioGL3-GL-3-GL3-Monoclonal/17-5711-82
CD4 PeCy7 https://www.thermofisher.com/antibody/product/CD4-Antibody-clone-GK1-5-Monoclonal/25-0041-82
CD8 APC-eFluor 780 https://www.thermofisher.com/antibody/product/CD8a-Antibody-clone-53-6-7-Monoclonal/47-0081-82
CD11c PerCpCy5.5 https://www.thermofisher.com/antibody/product/CD11c-Antibody-clone-N418-Monoclonal/45-0114-82
CD19 PeCy7 https://www.thermofisher.com/antibody/product/CD19-Antibody-clone-eBio1D3-1D3-Monoclonal/25-0193-82
CD11b PerCpCy5.5 https://www.thermofisher.com/antibody/product/CD11b-Antibody-clone-M1-70-Monoclonal/45-0112-82
CD11b APC https://www.thermofisher.com/antibody/product/CD11b-Antibody-clone-M1-70-Monoclonal/17-0112-82
CD11b AF488 https://www.thermofisher.com/antibody/product/CD11b-Antibody-clone-M1-70-Monoclonal/53-0112-82

F4/80 PeCy7 https://www.thermofisher.com/antibody/product/F4-80-Antibody-clone-BM8-Monoclonal/25-4801-82
CD206 FITC https://www.biolegend.com/fr-ch/products/fitc-anti-human-cd206-mmr-antibody-2993
anti-F4/80 https://www.scbt.com/p/f4-80-antibody-m-300
anti-F4/80 https://www.cellsignal.com/products/primary-antibodies/f4-80-d2s9r-xp-rabbit-mab/70076
anti-CD206 https://www.scbt.com/p/cd206-antibody-d-1
anti-CD11c https://www.cellsignal.com/products/primary-antibodies/cd11c-d1v9y-rabbit-mab/97585

Eukaryotic cell lines

Policy information about cell lines and Sex and Gender in Research

Cell line source(s)	ATTC (3T3-L1)
Authentication	Non of the cell lines were authenticated.
Mycoplasma contamination	Cell lines were not tested for mycoplasma contamination.
Commonly misidentified lines (See ICLAC register)	3T3 L1

Animals and other research organisms

Policy information about studies involving animals; ARRIVE guidelines recommended for reporting animal research, and Sex and Gender in Research

Laboratory animals	Mouse (GRLysmCre and GRClec4fCre mice), C57BL/6 background, HFD feeding for up to 29 weeks starting from age 8 to 12 weeks Mice were housed at a 12/12 dark/light cycle. Humidity was controlled to be between 55-65%. Ambient temperature was set to 22° C.
Wild animals	Study did not involve wild animals
Reporting on sex	All experiments were performed in male mice.
Field-collected samples	Study did not involve samples collected from the field.
Ethics oversight	All animal experiments were performed in accordance with accepted standards of animal welfare and with permission of the responsible authorities of the Thüringer Landesamt für Lebensmittelsicherheit und Verbraucherschutz and the Regierungspräsidium Tübingen (License 1436 and License 1332)

Note that full information on the approval of the study protocol must also be provided in the manuscript.

ChIP-seq

Data deposition

- [x] Confirm that both raw and final processed data have been deposited in a public database such as GEO.
- [x] Confirm that you have deposited or provided access to graph files (e.g. BED files) for the called peaks.

Data access links
May remain private before publication.

To review GEO accession GSE200371:
Go to https://www.ncbi.nlm.nih.gov/geo/query/acc.cgi?acc=GSE200371
Enter token wdwbocystbmzvof into the box

Files in database submission

GSE200371_RNA_ATM_DESeq2.txt.gz
GSM6032199 RNA_ATM_GRflox_1_R1.fq.gz
GSM6032199 RNA_ATM_GRflox_1_R2.fq.gz
GSM6032200 RNA_ATM_GRflox_2_R1.fq.gz
GSM6032200 RNA_ATM_GRflox_2_R2.fq.gz
GSM6032201 RNA_ATM_GRLysMCre_1_R1.fq.gz
GSM6032201 RNA_ATM_GRLysMCre_1_R2.fq.gz
GSM6032202 RNA_ATM_GRLysMCre_2_R1.fq.gz
GSM6032202 RNA_ATM_GRLysMCre_2_R2.fq.gz

GSE200371_RNA_Mac_DESeq2.txt.gz
GSM6032203 RNA_Mac_wt_veh_1_R1.fq.gz
GSM6032203 RNA_Mac_wt_veh_1_R2.fq.gz
GSM6032204 RNA_Mac_wt_veh_2_R1.fq.gz
GSM6032204 RNA_Mac_wt_veh_2_R2.fq.gz
GSM6032205 RNA_Mac_wt_dex_1_R1.fq.gz
GSM6032205 RNA_Mac_wt_dex_1_R2.fq.gz
GSM6032206 RNA_Mac_wt_dex_2_R1.fq.gz

GSM6032206 RNA_Mac_wt_dex_2_R2.fq.gz
GSM6032207 RNA_Mac_wt_il4_1_R1.fq.gz
GSM6032207 RNA_Mac_wt_il4_1_R2.fq.gz
GSM6032208 RNA_Mac_wt_il4_2_R1.fq.gz
GSM6032208 RNA_Mac_wt_il4_2_R2.fq.gz
GSM6032209 RNA_Mac_wt_il4dex_1_R1.fq.gz
GSM6032209 RNA_Mac_wt_il4dex_1_R2.fq.gz
GSM6032210 RNA_Mac_wt_il4dex_2_R1.fq.gz
GSM6032210 RNA_Mac_wt_il4dex_2_R2.fq.gz
GSM6032211 RNA_Mac_GRnull_veh_1_R1.fq.gz
GSM6032211 RNA_Mac_GRnull_veh_1_R2.fq.gz
GSM6032212 RNA_Mac_GRnull_veh_2_R1.fq.gz
GSM6032212 RNA_Mac_GRnull_veh_2_R2.fq.gz
GSM6032213 RNA_Mac_GRnull_dex_1_R1.fq.gz
GSM6032213 RNA_Mac_GRnull_dex_1_R2.fq.gz
GSM6032214 RNA_Mac_GRnull_dex_2_R1.fq.gz
GSM6032214 RNA_Mac_GRnull_dex_2_R2.fq.gz
GSM6032215 RNA_Mac_GRnull_il4_1_R1.fq.gz
GSM6032215 RNA_Mac_GRnull_il4_1_R2.fq.gz
GSM6032216 RNA_Mac_GRnull_il4_2_R1.fq.gz
GSM6032216 RNA_Mac_GRnull_il4_2_R2.fq.gz
GSM6032217 RNA_Mac_GRnull_il4dex_1_R1.fq.gz
GSM6032217 RNA_Mac_GRnull_il4dex_1_R2.fq.gz
GSM6032218 RNA_Mac_GRnull_il4dex_2_R1.fq.gz
GSM6032218 RNA_Mac_GRnull_il4dex_2_R2.fq.gz

GSE200371_H3K27ac_Mac_DEseq2.txt.gz
GSM6032219 H3K27ac_Mac_GRflox_1_R1.fq.gz
GSM6032219 H3K27ac_Mac_GRflox_1_R2.fq.gz
GSM6032220 H3K27ac_Mac_GRflox_2_R1.fq.gz
GSM6032220 H3K27ac_Mac_GRflox_2_R2.fq.gz
GSM6032221 H3K27ac_Mac_GRflox_3_R1.fq.gz
GSM6032221 H3K27ac_Mac_GRflox_3_R2.fq.gz
GSM6032222 H3K27ac_Mac_GRLysMCre_1_R1.fq.gz
GSM6032222 H3K27ac_Mac_GRLysMCre_1_R2.fq.gz
GSM6032223 H3K27ac_Mac_GRLysMCre_2_R1.fq.gz
GSM6032223 H3K27ac_Mac_GRLysMCre_2_R2.fq.gz
GSM6032224 H3K27ac_Mac_GRLysMCre_3_R1.fq.gz
GSM6032224 H3K27ac_Mac_GRLysMCre_3_R2.fq.gz

Genome browser session
(e.g. UCSC)

https://genome.ucsc.edu/s/dr.arauch/Ulm

Methodology

Replicates

RNA-seq experiments have been replicated twice and ChIP-seq experiments were replicated three times, all of them with cells from independent mice. Replicate agreement was determined by performing a Pearson's correlation over the read density values.

RNA-seq ATM based on on tag density in gene bodies from GSE200371_RNA_ATM_DESeq2.txt.gz: GRflox (GSM6032199 - GSM6032200: 0.984), GRLysMCre (GSM6032201 - GSM6032202: 0.954)

RNA-seq Mac based on on tag density in gene bodies from GSE200371_RNA_Mac_DESeq2.txt.gz: wt veh (GSM6032203 - GSM6032204: 0.998), wt dex (GSM6032205 - GSM6032206: 0.986), wt il4 (GSM6032207 - GSM6032208: 0.989), wt il4 + dex (GSM6032209 - GSM6032210: 0.996), GRnull veh (GSM6032211 - GSM6032212: 0.988), GRnull dex (GSM6032213 - GSM6032214: 0.994), GRnull il4 (GSM6032215 - GSM6032216: 0.996), GRnull il4 + dex (GSM6032217 - GSM6032218: 0.996)

Correlation H3K27ac ChIP-seq based on tag density in peak file coordinates in GSE200371_H3K27ac_Mac_DEseq2.txt.gz: GRflox (GSM6032219 - GSM6032220: 0.993; GSM6032219 - GSM6032221: 0.985; GSM6032220 - GSM6032221: 0.994), GRLysMCre (GSM6032222 - GSM6032223: 0.996; GSM6032222 - GSM6032224: 0.994; GSM6032223 - GSM6032224: 0.994)

Sequencing depth

All Samples were paired end sequencing
Sample; Sequencing depth; Uniquely aligned; fragmentLengthEstimate (STAR)

GSM6032199 RNA_ATM_GRflox_1; 20,242,533; 15,913,580; 142
GSM6032200 RNA_ATM_GRflox_2; 31,852,360; 25,253,069; 143
GSM6032201 RNA_ATM_GRLysMCre_1; 21,073,067; 16,861,514; 148
GSM6032202 RNA_ATM_GRLysMCre_2; 22,389,277; 17,331,915; 146

GSM6032203 RNA_Mac_wt_veh_1; 27,690,983; 23,933,181; 639
GSM6032204 RNA_Mac_wt_veh_2; 22,071,264; 18,982,910; 764
GSM6032205 RNA_Mac_wt_dex_1; 22,661,812; 19,759,681; 463
GSM6032206 RNA_Mac_wt_dex_2; 23,924,882; 20,673,411; 601
GSM6032207 RNA_Mac_wt_il4_1; 17,139,658; 14,524,116; 783
GSM6032208 RNA_Mac_wt_il4_2; 24,455,574; 21,106,762; 453
GSM6032209 RNA_Mac_wt_il4dex_1; 17,801,555; 15,465,806; 608
GSM6032210 RNA_Mac_wt_il4dex_2; 25,782,272; 22,361,065; 463
GSM6032211 RNA_Mac_GRnull_veh_1; 20,640,739; 17,948,862; 619
GSM6032212 RNA_Mac_GRnull_veh_2; 21,908,933; 18,837,070; 594
GSM6032213 RNA_Mac_GRnull_dex_1; 22,586,027; 19,556,134; 190
GSM6032214 RNA_Mac_GRnull_dex_2; 21,913,666; 18,922,775; 740
GSM6032215 RNA_Mac_GRnull_il4_1; 19,565,154; 16,798,108; 797
GSM6032216 RNA_Mac_GRnull_il4_2; 23,975,954; 20,699,624; 654
GSM6032217 RNA_Mac_GRnull_il4dex_1; 17,002,299; 14,654,148; 631
GSM6032218 RNA_Mac_GRnull_il4dex_2; 25,658,955; 22,021,047; 578

GSM6032219 H3K27ac_Mac_GRflox_1; 19,811,337; 17,541,935; 217
GSM6032220 H3K27ac_Mac_GRflox_2; 17,751,958; 15,490,952; 213
GSM6032221 H3K27ac_Mac_GRflox_3; 5,060,117; 4,559,578; 225
GSM6032222 H3K27ac_Mac_GRLysMCre_1; 11,033,297; 9,862,429; 235
GSM6032223 H3K27ac_Mac_GRLysMCre_2; 14,467,203; 13,113,455; 221
GSM6032224 H3K27ac_Mac_GRLysMCre_3; 20,575,555; 18,095,044; 217

Antibodies

Mouse monoclonal H3K27ac antibody Active Motif #39685; Clone MABI 0309; RRID: AB_2793305

Peak calling parameters

```
# Find peaks with histone option
findPeaks H3K27ac_WT_1_star.Aligned.out.TD/ -style histone -o WT_1.txt
findPeaks H3K27ac_WT_2_star.Aligned.out.TD/ -style histone -o WT_2.txt
findPeaks H3K27ac_WT_3_star.Aligned.out.TD/ -style histone -o WT_3.txt
findPeaks H3K27ac_KO_1_star.Aligned.out.TD/ -style histone -o KO_1.txt
findPeaks H3K27ac_KO_2_star.Aligned.out.TD/ -style histone -o KO_2.txt
findPeaks H3K27ac_KO_3_star.Aligned.out.TD/ -style histone -o KO_3.txt

# merge wt replicates (keep only those in all three replicates)
mergePeaks WT_*.txt -d given -prefix Merge_WT

# merge ko replicates (keep only those in all three replicates)
mergePeaks KO_*.txt -d given -prefix Merge_KO

# merge wt and ko file, make to bed file and remove blacklisted peaks
mergePeaks Merge_WT_WT_1.txt_WT_2.txt_WT_3.txt Merge_KO_KO_1.txt_KO_2.txt_KO_3.txt > Peaks_H3K27ac.txt
awk '{print $2"\t"$3"\t"$4"\t"$1}' Peaks_H3K27ac.txt > Peaks_H3K27ac_tmp.bed
sed '1d' Peaks_H3K27ac_tmp.bed > Peaks_H3K27ac.bed
bedtools intersect -v -a Peaks_H3K27ac.bed -b mm10-blacklist.v2.bed > Peaks_H3K27ac_blacklisted.bed

# Annotate TagDir for diff H3K27ac levels
annotatePeaks.pl Peaks_H3K27ac_blacklisted.bed mm10 -noadj -d H3K27ac_WT*.TD H3K27ac_KO*.TD > TagCounts_H3K27ac.txt
```

Data quality

IP-efficiency (tags in peaks compared to background) was used as quality measurement and Pearson's correlation for read density within peak region was used to control replicates (see above)
GSM6032219 H3K27ac_Mac_GRflox_1: 9,40 %
GSM6032220 H3K27ac_Mac_GRflox_2: 7.72 %
GSM6032221 H3K27ac_Mac_GRflox_3: 12.32 %
GSM6032222 H3K27ac_Mac_GRLysMCre_1: 10.48 %
GSM6032223 H3K27ac_Mac_GRLysMCre_2: 11.46 %
GSM6032224 H3K27ac_Mac_GRLysMCre_3: 7.26 %

Software

HOMER http://homer.salk.edu/homer/
Bedtools http://bedtools.readthedocs.io/en/latest/
STAR aligner https://github.com/alexdobin/STAR

Flow Cytometry

Plots

Confirm that:

- [x] The axis labels state the marker and fluorochrome used (e.g. CD4-FITC).
- [x] The axis scales are clearly visible. Include numbers along axes only for bottom left plot of group (a 'group' is an analysis of identical markers).
- [x] All plots are contour plots with outliers or pseudocolor plots.
- [x] A numerical value for number of cells or percentage (with statistics) is provided.

Methodology

Sample preparation	SVF or co-cultures were harvested as described in the methods section. After erylysis, cells were resuspended in FACS buffer (PBS 2% FCS) and counted. Cell surface antigens were blocked with Anti-Mouse CD16/CD32 (14-0161, eBioscience) and stained, details for each antibody can be found in the methods. Single stained controls were used for compensation.
Instrument	Canto II or LSR II
Software	BD FACS Diva was used for acquisition, FlowJo was used for analysis and Graphpad Prism for statistics.
Cell population abundance	Macrophages, the main population of interest were around 15% of all live cells. Details of individual populations can be found in the manuscript.
Gating strategy	SSC-A FSC-A was used to identify cells, singlets were identified using FSC-H and FSC-A. DAPI was used to exclude dead cells. Macrophages were defined as CD11b+ F4/80+ with polarisation markers CD206 and CD11c. Dendritic cells were identified as CD11b+ F4/80- CD11c+ cells. B cells defined as TCR-b- CD-19+. T cells as: TCR-b+ followed by CD8 and CD4+.

- [x] Tick this box to confirm that a figure exemplifying the gating strategy is provided in the Supplementary Information.
